# Serotonin Syndrome Induced by Fentanyl Alone in an Adult Patient After Cardiac Surgery: A Case Report

**DOI:** 10.7759/cureus.64832

**Published:** 2024-07-18

**Authors:** Nobutoshi Matsumura, Yoshio Nitta, Tomoyuki Endo, Takafumi Kobayashi, Seijiro Yoshida

**Affiliations:** 1 Department of Cardiovascular Surgery, Sendai City Medical Center, Sendai, JPN; 2 Division of Emergency and Disaster Medicine, Tohoku Medical and Pharmaceutical University, Sendai, JPN; 3 Department of Anesthesiology, Sendai City Medical Center, Sendai, JPN

**Keywords:** critical care, cardiac surgery, dantrolene, fentanyl, serotonin toxicity, serotonin syndrome

## Abstract

Serotonin syndrome is a rare but potentially fatal condition characterized by altered mental status, autonomic hyperactivity, and neuromuscular abnormalities. Although fentanyl is known to be a causative agent of serotonin syndrome, most reports have shown that fentanyl-related serotonin syndrome is caused by multiple drug interactions, and only one case of serotonin syndrome caused by fentanyl alone has been reported in a pediatric patient. In this report, we describe a case of postoperative serotonin syndrome caused by fentanyl alone in an adult patient after cardiac surgery. A 66-year-old male was diagnosed with unstable angina pectoris and underwent off-pump coronary artery bypass grafting. Two hours after the intensive care unit (ICU) admission, he exhibited symptoms of sweating, tremors, and muscle rigidity. Four hours later, the body temperature rose to 40.0 °C, suggesting malignant hyperthermia or a similar condition. Dantrolene was administered to the patient, and all symptoms improved within several minutes. However, the patient experienced a relapse of symptoms every four to six hours, requiring additional dantrolene treatment each time. Although no other serotonergic agents were used, we suspected serotonin syndrome induced by fentanyl alone and discontinued its use on postoperative day three. Following the discontinuation of fentanyl, no further episodes were observed. The patient was discharged from the hospital without any complications on postoperative day 29. During a subsequent check-up, the patient was found to have a sternal dehiscence and underwent one-stage sternal reconstruction. General anesthesia was induced and maintained without the use of fentanyl. The patient was discharged 10 days after surgery without symptoms of serotonin syndrome. In a patient with postoperative hyperthermia and neuromuscular abnormalities, serotonin syndrome should be considered when fentanyl is administered. Dantrolene may be beneficial in managing serotonin syndrome caused by fentanyl alone and/or benzodiazepine resistance.

## Introduction

Serotonin syndrome is a rare but potentially fatal condition with a triad of altered mental status, autonomic hyperactivity, and neuromuscular abnormalities [1]. This condition arises from the overstimulation of serotonin receptors in the central nervous system due to certain drugs or their interactions [1]. Fentanyl, a synthetic opioid widely used for pain management, is a 5-HT_1A_ agonist and also has a serotonin reuptake inhibition effect, synergistically raising intrasynaptic serotonin levels [2,3]. Although fentanyl is known to be a causative agent of serotonin syndrome, most reports to date have shown that fentanyl-related serotonin syndrome is caused by multiple drug interactions [4,5], and to the best of our knowledge, only one case of serotonin syndrome caused by fentanyl alone has been reported in a pediatric patient [6]. Herein, we describe a case of postoperative serotonin syndrome caused by fentanyl alone in an adult patient who underwent cardiac surgery.

## Case presentation

A 66-year-old Asian male with a height of 167.5 cm and body weight of 81.7 kg presented to our hospital. He reported a significant increase in the frequency of chest pain over the past month. His medical history included previous myocardial infarction, hypertension, and dyslipidemia. He was taking aspirin, clopidogrel, esomeprazole, bisoprolol, candesartan, and pitavastatin without any antipsychotic drugs or serotonergic agonists. His family history was unremarkable, with no instances of malignant hyperthermia. Upon physical examination, the thyroid was not palpable, and blood tests revealed normal thyroid hormone levels (Table 1).

**Table 1:** Preoperative blood test WBC, white blood cell; RBC, red blood cell; Hb, hemoglobin; Hct, hematocrit; Plt, platelet; MCV, mean corpuscular volume; MCH, mean corpuscular hemoglobin; APTT, activated partial thromboplastin time; PT-INR, prothrombin time-international normalized ratio; TP, total protein; Alb, albumin; T-bil, total bilirubin; AST, aspartate aminotransferase; ALT, alanine aminotransferase; ALP, alkaline phosphatase; γGT, γ-glutamyl transpeptidase; LD, lactate dehydrogenase; BUN, blood urea nitrogen; Cre, Creatinine; CK, creatine kinase; CK-MB, creatine kinase-myoglobin binding; CRP, C-reactive protein; THS, thyroid-stimulating hormone

Laboratory parameter	Value	Reference range
Complete blood count		
WBC (/µL)	9710	3000-9000
RBC (x10^4^/µL)	411	430-560
Hb (g/dL))	12.9	13.7-17.4
Hct (%)	38.3	40.2-51.5
Plt (x10^4^/µL)	27.3	13.1-36.5
MCV (fl)	93	83-101
MCH (pg)	31.4	28.1-34.5
Coagulation factors		
APTT (sec)	34	25.1-36.5
PT-INR	1.02	n/a
D-dimer (µg/mL)	1.18	0.00-1.00
Blood chemistry		
TP (g/dL)	6.1	6.5-8.3
Alb (g/dL)	3.3	3.9-4.9
T-bil (mg/dL)	0.4	0.2-1.2
AST (U/L)	17	7-38
ALT (U/L)	19	4-44
ALP (U/L)	334	106-345
γGT (U/L)	253	11-58
LD (U/L)	168	106-220
BUN (mg/dL)	13.7	7.8-22.0
Cre (mg/dL)	1.21	0.60-1.10
Na (mEq/L)	140.5	135.0-147.0
K (mEq/L)	3.9	3.3-4.8
Cl (mEq/L)	106.7	98.0-108.0
Ca (mg/dL)	6.7	8.8-10.2
CK (U/L)	154	0-250
CK-MB (ng/mL)	0.9	0.0-7.2
CRP (mg/dL)	0.7	0.0-0.3
TSH (µIU/mL)	1.36	0.61-4.23
Free T3 (pg/mL)	3.12	1.68-3.67
Free T4 (ng/mL)	1.13	0.70-1.48

Coronary angiography showed significant stenosis in the left main coronary trunk, leading to a diagnosis of unstable angina pectoris. The patient underwent off-pump coronary artery bypass grafting. General anesthesia was induced with fentanyl, propofol, and rocuronium and maintained with fentanyl (total of 1000 µg in the surgery), remifentanil, morphine, propofol, and rocuronium. The surgery was completed without any significant complications, and the patient was admitted to the intensive care unit (ICU) under mechanical ventilation. The blood test at ICU admission was reasonable as postoperative changes (Table 2).

**Table 2:** Postoperative blood test WBC, white blood cell; RBC, red blood cell; Hb, hemoglobin; Hct, hematocrit; Plt, platelet; MCV, mean corpuscular volume; MCH, mean corpuscular hemoglobin; APTT, activated partial thromboplastin time; PT-INR, prothrombin time-international normalized ratio; TP, total protein; Alb, albumin; T-bil, total bilirubin; AST, aspartate aminotransferase; ALT, alanine aminotransferase; ALP, alkaline phosphatase; γGT, γ-glutamyl transpeptidase; LD, lactate dehydrogenase; BUN, blood urea nitrogen; Cre, Creatinine; CK, creatine kinase; CK-MB, creatine kinase-myoglobin binding; CRP, C-reactive protein

Laboratory parameter	Value	Reference range
Complete blood count		
WBC (/µL)	10610	3000-9000
RBC (x10^4^/µL)	336	430-560
Hb (g/dL))	10.1	13.7-17.4
Hct (%)	30.2	40.2-51.5
Plt (x10^4^/µL)	23.4	13.1-36.5
MCV (fl)	90	83-101
MCH (pg)	30.1	28.1-34.5
Coagulation factors		
APTT (sec)	32.4	25.1-36.5
PT-INR	1.17	n/a
D-dimer (µg/mL)	3.76	0.00-1.00
Blood chemistry		
TP (g/dL)	4.8	6.5-8.3
Alb (g/dL)	2.8	3.9-4.9
T-bil (mg/dL)	0.3	0.2-1.2
AST (U/L)	19	7-38
ALT (U/L)	17	4-44
ALP (U/L)	209	106-345
γGT (U/L)	16	11-58
LD (U/L)	153	106-220
BUN (mg/dL)	14.9	7.8-22.0
Cre (mg/dL)	1.21	0.60-1.10
Na (mEq/L)	144.2	135.0-147.0
K (mEq/L)	3.8	3.3-4.8
Cl (mEq/L)	110.9	98.0-108.0
Ca (mg/dL)	7.5	8.8-10.2
CK (U/L)	191	0-250
CK-MB (ng/mL)	1.9	0.0-7.2
CRP (mg/dL)	0.6	0.0-0.3

**Figure 1:**
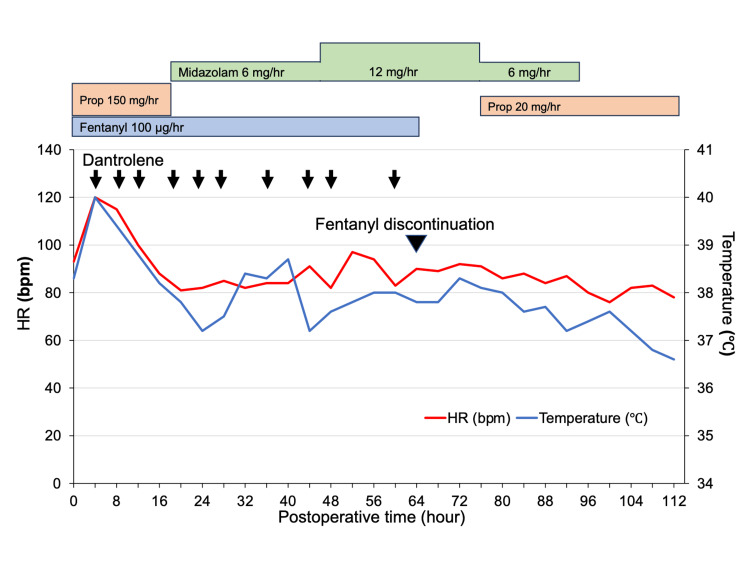
Clinical course in the intensive care unit Body temperature was measured in the bladder. Prop, propofol

The continuous intravenous administrations of fentanyl (100 µg/hour) and propofol were used as analgesic and sedative, respectively, without muscular relaxants. The clinical course in the ICU is shown in Figure 1.

Two hours after ICU admission (eight hours after the first fentanyl infusion), the patient's partial pressure of carbon dioxide increased from 37.6 to 54.6 mmHg without any changes in ventilator settings. At the same time, he experienced sweating, tremors, and muscle rigidity in his masseter and extremities. Despite the administration of 10 mg of diazepam twice, his symptoms did not improve. By this point, a total of 1200 µg of fentanyl had been used. Four hours after ICU admission, the patient's bladder temperature rose to 40.0 °C, and his heart rate increased to 120 bpm. The patient in question did not have a family history of malignant hyperthermia, and no inhalation anesthetics or depolarizing muscle relaxants were administered during the surgery. Despite this, the patient exhibited clinical manifestations that suggested malignant hyperthermia or a similar condition. Dantrolene (1 mg/kg) was administered to the patient, and all symptoms improved within several minutes. However, the patient experienced a relapse of tremors and muscle rigidity in the masseter and extremities every four to six hours, requiring additional dantrolene treatment each time. Twenty hours after ICU admission, propofol was replaced to midazolam but the neuromuscular abnormalities persisted. Although no other serotonergic agents were used, we suspected serotonin syndrome induced by fentanyl alone and discontinued its use on postoperative day three. Following the discontinuation of fentanyl, no further episodes of neuromuscular abnormalities were observed. The use of dantrolene did not result in any notable elevation of serum creatine kinase or myoglobin, considering the damage caused by the surgery (Table 3).

**Table 3:** Postoperative serum creatinine kinase and myoglobin ICU indicates intensive care unit; POD, postoperative day; n/a, not available

	ICU admission	POD 1	POD 2	POD 3	POD 4	POD 5
Creatinine kinase (U/mL)	191	541	445	253	192	146
Myoglobin (ng/mL)	165	533	388	127	n/a	n/a

The patient was successfully extubated on postoperative day seven and discharged from the ICU on postoperative day 13. The patient was discharged from the hospital without any complications on postoperative day 29.

One year and five months after the initial surgery, the patient was found to have a sternal dehiscence during a regular check-up. As there were no signs of mediastinal infection, the patient underwent one-stage sternal reconstruction. General anesthesia was induced with ketamine, propofol, and rocuronium, and maintained with ketamine, morphine, propofol, and rocuronium. The operation was completed without any complications, and the patient was discharged home 10 days after surgery without any symptoms of serotonin syndrome.

## Discussion

Serotonin syndrome is a potentially life-threatening condition that can result in hyperthermia and neuromuscular abnormalities during the perioperative period. It is essential to consider other conditions in the differential diagnosis, including malignant hyperthermia, malignant syndrome, and thyroid storm [1]. Malignant hyperthermia is an inherited skeletal muscle disease that occurs in response to anesthetics, which abnormally enhance the calcium release mechanism from the sarcoplasmic reticulum, a calcium storage compartment in skeletal muscle cells, into the cytoplasm [7]. In this case, malignant hyperthermia was ruled out since the patient did not have a family history of malignant hyperthermia and was not administered inhalation anesthetics or depolarizing muscle relaxants. Malignant syndromes are triggered by dopamine antagonists such as antipsychotics and develop over several days to several weeks [8]. In the present case, antipsychotic medication was not prescribed to the patient and the onset was acute occurring two hours after the surgery. A thyroid storm was also ruled out based on the absence of a palpable thyroid gland and normal thyroid function during the preoperative examination. The diagnosis of serotonin syndrome was made after considering the use of fentanyl, the drug responsible for the condition, both intra- and postoperatively, and the recurrence that ceased when the medication was discontinued. Moreover, the patient did not experience serotonin syndrome when he underwent sternal reconstruction surgery without fentanyl.

Fentanyl-induced serotonin syndrome has been reported to be associated with drug interactions with other causative agents such as selective serotonin reuptake inhibitors [4,5], and only one case of serotonin syndrome caused by fentanyl alone has been reported in a pediatric patient [6]. As the mechanism of action, fentanyl synergistically increases intrasynaptic serotonin levels through its 5-HT1A agonist action and serotonin reuptake inhibition effect. Although fentanyl seems to induce serotonin syndromes more frequently based on this synergistic mechanism, the shorter half-life of fentanyl (3.6 hours) compared to other serotonergic agonists may be the reason it does not cause serotonin syndrome when used with bolus administration alone or a low dose of continuous infusion [9]. In the current case, the bolus and continuous intravenous infusion during and after surgery (1000 µg and 200 µg, respectively, by the onset) may have been a factor in the onset of serotonin syndrome. Moreover, since nondepolarizing muscle relaxants are used for the treatment of hyperthermia (more than 41.1 °C) in patients with serotonin syndrome, rocuronium that had been used in the surgery (total of 200 mg) might delay the onset of neuromuscular abnormalities [1]. When symptoms such as hyperthermia and neuromuscular abnormalities occur after high-dose fentanyl infusion, serotonin syndrome should be suspected, even if fentanyl is used alone without other serotonergic agents.

Prompt treatment is essential once serotonin syndrome has been diagnosed. The first course of action is to discontinue the causative agent, as was done in this case with the discontinuation of fentanyl. When the condition is severe and requires intervention, cyproheptadine, a serotonin receptor antagonist, and/or benzodiazepines should be administered [10]. However, in this instance, cyproheptadine was unavailable. Intravenous benzodiazepines, such as diazepam and midazolam, were administered but did not alleviate the symptoms. The only effective treatment in this case was dantrolene, which prevented the elevation of serum creatine kinase and myoglobin. Although dantrolene has no serotonergic action and is generally considered pharmacologically ineffective, some reports have shown its usefulness [11-13]. Dantrolene is a direct-acting skeletal muscle relaxant that blocks calcium release from intracellular storage in the sarcoplasmic reticulum [14]. In addition to its use in treating malignant hyperthermia and malignant syndrome, oral dantrolene is also used to treat spasticity. Therefore, dantrolene may be effective in treating neuromuscular abnormalities in serotonergic syndrome caused by fentanyl alone and/or with benzodiazepine resistance. Given the difficulty in differentiating between perioperative hyperthermia and neuromuscular abnormalities, dantrolene may be useful to provide time for an accurate diagnosis for patients in severe conditions and who are benzodiazepine resistant.

## Conclusions

We documented a case of serotonin syndrome induced by fentanyl alone in an adult patient following cardiac surgery. In a patient with postoperative hyperthermia and neuromuscular abnormalities, it is essential to differentiate between serotonin syndrome and other conditions, such as malignant hyperthermia, malignant syndrome, and thyroid storm. When fentanyl is used at high doses, even without other serotonin-related agents, serotonin syndrome should be considered. Dantrolene may be beneficial in addressing neuromuscular abnormalities associated with serotonin syndrome caused by fentanyl alone and/or benzodiazepine resistance.
